# Enhancing implementation of information and communication technologies for post-discharge care among hospitalized older adult patients: development of a multifaceted implementation intervention package using the behavior change wheel and implementation research logic model

**DOI:** 10.1186/s43058-025-00739-4

**Published:** 2025-05-01

**Authors:** Dorothy Yingxuan Wang, Eliza Lai-Yi Wong, Annie Wai-Ling Cheung, Zoe Pui-Yee Tam, Kam-Shing Tang, Eng-Kiong Yeoh

**Affiliations:** 1 JC School of Public Health and Primary Care, Faculty of Medicine, The Chinese University of Hong Kong, Hong Kong SAR, China; 2 Centre for Health Systems & Policy Research, JC School of Public Health and Primary Care, Faculty of Medicine, The Chinese University of Hong Kong, Hong Kong SAR, China; 3 Kwong Wah Hospital, Hospital Authority, Hong Kong SAR, China

**Keywords:** Implementation intervention, Information technology, Self care, Patient discharge, Hospital to home

## Abstract

**Background:**

The integration of information and communication technologies in clinical practice can supplement traditional care pathways on discharge education and has exhibited evident benefits in improving patient health outcomes. However, healthcare providers have reported difficulties in adopting such technologies. The existing evidence on implementation interventions supporting the implementation of information and communication technologies is insufficient, characterized by infrequent utilization or reporting of implementation theories in implementation intervention designs. This study aims to outline the creation of a theory-informed implementation intervention package to enhance the clinical implementation of information and communication technologies for post-discharge self-care among hospitalized older adults.

**Methods:**

This study systematically applies the Behavior Change Wheel (BCW) approach, involving behavior diagnosis, identification of intervention options, and intervention content selection, informed by conceptual frameworks, empirical data, and relevant literature. Additionally, the Implementation Research Logic Model is utilized to synthesize, organize, and visually present the collected data.

**Results:**

This structured process identified and selected five intervention functions, 11 behavior change techniques, and four policy categories. A multifaceted implementation intervention package was developed, containing four components: (i) flexible and sustainable training, (ii) mass media and opinion leader campaign, (iii) technology and workflow redesign, and (iv) regular corporate-level audit and feedback.

**Conclusions:**

The study addresses the incomplete evidence base for implementation interventions supporting clinical information and communication technology implementation, presenting a practical, evaluable, and scalable theory-informed implementation intervention package. By providing an example of the application of the BCW approach and logic model, this study contributes to the knowledge on implementation intervention design, offering valuable insights for researchers and practitioners aiming to improve healthcare providers' behavior change and post-discharge care management with technology-based interventions.

**Supplementary Information:**

The online version contains supplementary material available at 10.1186/s43058-025-00739-4.

Contributions to the literature • This study addresses the incomplete evidence base for implementation interventions supporting clinical information and communication technology implementation, which can be generalized to similar contexts aiming to improve clinicians' behavior change and post-discharge care with technology-based interventions.

• The meticulous documentation of various intervention options and rationale for selecting and excluding with standardized appraisal criteria allowed the transparency and generalizability of our implementation intervention development methods.

• This study promotes the application of the combination of implementation frameworks in implementation intervention designs and facilitates the comparison of the benefits derived from implementation interventions with and without implementation theories.

## Background

The period of transitioning from hospitals to home is marked by increased risks of drug-related readmissions [1–3], highlighting the importance of promoting post-discharge medication self-management among older adults [4]. Incorporating information and communication technologies, such as Electronic Health Record-based interventions, Clinical Decision Support System, mHealth and eHealth, into inpatient settings has demonstrated the potential to enhance care quality and efficiency [5]. Information and communication technology adoption can supplement traditional care pathways in discharge education [6], enabling effective patient-provider communication regarding discharge instructions [7, 8]. Nonetheless, studies suggest that health providers often encounter difficulties implementing information and communication technologies [9]. Merely providing health providers with technologies is insufficient to bring about the envisioned healthcare transformation [10]. Integrating information and communication technologies into patient care pathways necessitates changes in health providers’ practices [11]. The process of practice change is influenced by various intrinsic and extrinsic factors such as individual health professional and patient related factors, professional interactions, organizational capacity to change, and social and political factors, as evidenced by a comprehensive review [12]. The current evidence base for implementation interventions targeting information and communication technologies remains incomplete [13], with haphazard and poorly specified designs [14], minimal attention to changes across individual, organizational, and system levels [15], and seldomly employed or reported theories in such implementation intervention design [15].

Behavior change is more likely to occur when decisions regarding implementation intervention content adhere to systematic guidelines. Behavior Change Wheel (BCW) serves as a systematic approach explicitly listing each step of implementation intervention design, encompassing understanding the behavior that needs to be changed, identifying implementation intervention options, and determining the implementation intervention content [16]. In addition, BCW provides a theory-driven approach to link the choice of implementation interventions to relevant determinants of change. Tailored implementation interventions targeting barriers to change have been associated with improved professional behavior [17], which is often overlooked or not reported [18]. While the BCW provides a framework for implementation intervention development, it requires judgment to determine the most suitable approach for a given context [16]. Explicitly describing the implementation intervention and its anticipated effects facilitates assessments necessary for understanding how implementation interventions work and predict outcomes [19]. Developing a pragmatic theory of change helps comprehend how an implementation intervention and its components will bring about successful implementation. However, there is a lack of utilization of the theory of change, program theory, or logic model [20]. The Implementation Research Logic Model (IRLM) can enhance the rigor and transparency of implementation studies by providing a common structure and specifying links and pathways for testing theories [21].

## Information and communication technology implementation in Hong Kong

Patient experience surveys conducted in Hong Kong have shed light on the suboptimal provision of comprehensive discharge information, especially regarding medication side effects [22]. These challenges are not unique to Hong Kong and have been observed in other countries [23–27]. The existing evidence base highlights various types of interventions designed to improve the transfer of discharge medication information and enhance patient recall. These interventions employ different methods, either singularly or in combination, including verbal, written, and technology-based approaches [28]. Examples include audio or visual materials, mobile applications or tablets, discharge summary letters, written medication management plan, and oral consultations provided by professionals such as pharmacists, physicians, and nurses [29].

In Hong Kong, in response to this issue, the Hospital Authority, which is a statutory body to manage Hong Kong's public hospitals, specialist clinics, and outpatient facilities, developed an information and communication technology called the post-discharge information summary (PDIS) in 2017. The aim of the PDIS is to enhance patient-provider communication on discharge medications and empower patients to self-manage medication side effects after discharge home. Our research team also had prior involvement in the development of the PDIS, as we led the annual patient experience survey commissioned by the Hospital Authority. Through this survey, we identified opportunities to improve the provision of discharge information, particularly regarding medication side effects [30]. The PDIS includes a salient medication reminder, an online drug database, and future follow-up appointments. The salient medication reminder was validated through Delphi surveys and discussions among 13 geriatric medical experts, generating a database covering 80% of the prescribed discharge medications alongside 235 most relevant and important side effects and warning signal items adapted to local older adult patients [31]. To ensure readability, the content is translated into Chinese and displayed in larger font sizes. The PDIS system is integrated into the electronic health record, enabling the generation of personalized discharge information. In 2018, the PDIS was piloted in four public tertiary hospitals and has gradually expanded to over 30 public hospitals in Hong Kong. Nurses designated as front-line implementers received pre-implementation training on how to use the system. They are required to print the written PDIS form and explain the content, including side effects and follow-up information, to patients or their caregivers upon discharge. Teach-back is not required. However, a staff survey among the four pilot hospitals conducted by our research team revealed inconsistent use of the PDIS, with only 78% of nurses consistently printing PDIS forms and 57% consistently explaining the content [32]. Therefore, two behavior targets were found: (i) to encourage nurses’ uptake of the PDIS and (ii) to support nurses in explaining PDIS information to each patient upon discharge.

Our research team conducted a comprehensive implementation evaluation project to identify the implementation barriers and facilitators and develop corresponding implementation interventions to enhance the PDIS implementation [33]. This project has assisted in identifying barriers and enablers that hinder or facilitate progress within this specific context, serving as the foundation for the implementation intervention design discussed in this paper [34]. Key facilitators included knowledge and skills, beliefs about the capability to implement the PDIS, beliefs about the value of the PDIS, willingness to implement, and goal setting. Key barriers included professional role, memory and decision processes, and environmental context and resources.

This study is part of this large implementation evaluation project, aiming to explicitly outline the steps taken to create a theory-informed multifaceted implementation intervention package to enhance health providers’ adherence to implementing the PDIS for post-discharge self-management among hospitalized older adults. This implementation intervention package aimed to be practical, evaluable, and scalable, utilizing a combination of conceptual frameworks, empirical data, relevant literature, and a logic model to gain a comprehensive understanding of the context and inform the selection of appropriate implementation interventions.

### Methods

## Theoretical frameworks used to develop the implementation intervention package

**Fig. 1:**
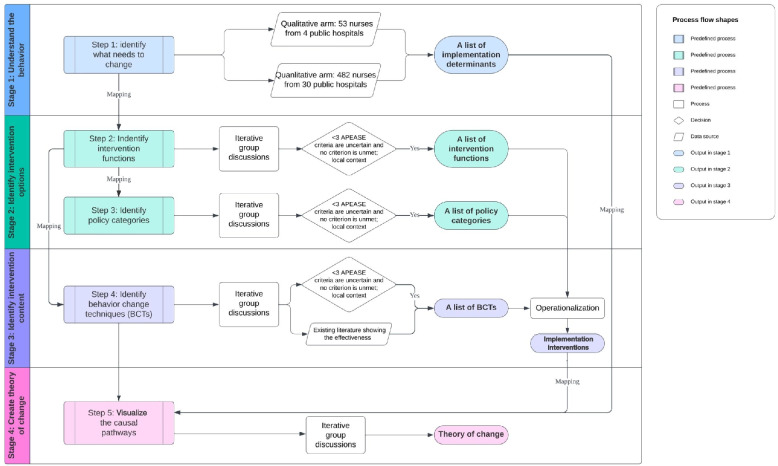
PDIS implementation intervention package design process. Abbreviations: PDIS: Post-discharge Information Summary; APEASE: Affordability, Practicability, Effectiveness, Acceptability, Side effects/safety, Equity

The design of the implementation intervention package was multistage (Fig. 1), primarily grounded in the BCW and its Guide to Designing Interventions [16]. The BCW is a comprehensive framework integrating 19 behavior change theories. At the hub of the BCW is the COM-B model, encompassing three key components: capability, opportunity, and motivation. To further explore the implementation determinants, the Theoretical Domains Framework (TDF) is applied [35, 36]. The TDF, derived from 33 theories and 84 constructs, provides a comprehensive framework with 14 domains, each corresponding to a specific COM-B component. The second layer of the BCW consists of nine intervention functions aiming to address the COM-B and TDF-informed determinants. The outer layer consists of seven policy categories supporting the delivery of intervention functions. The behavior change techniques were identified to further enact the selected intervention functions. behavior change techniques are the active ingredients of interventions, allowing them to be evaluated and replicated [37]. Finally, IRLM is applied to visualize the causal pathways between implementation determinants, implementation interventions, mechanisms of action, and implementation outcomes [21]. This study was approved by the Joint Chinese University of Hong Kong – New Territories East Cluster Clinical Research Ethics Committees (CREC Ref: 2019.436). The Standard for Reporting Implementation Studies (STaRI) was applied to report the results [38] (see Additional file 1).

## Data collection (stage 1): understand the behavior

A mixed-method study was conducted to investigate the factors influencing PDIS implementation among nurses [39]. The qualitative arm of the study recruited 53 nurses from the four pilot hospitals, participating in TDF-informed semi-structured interviews. The participants'statements were deductively coded into 14 TDF domains, and belief statements were generated within each domain. During the interview phase, it was observed that the PDIS system had expanded to over 30 hospitals across different geographical areas of Hong Kong. To enhance the generalizability of the qualitative findings, a quantitative arm was designed, employing a cross-sectional survey methodology. The survey instrument was developed based on the qualitative results obtained earlier. A total of 482 nurses completed the survey, providing their perceptions regarding the agreement and importance of each implementation determinant. The regression analysis identified nine TDF domains as potential significant predictors associated with an increased likelihood of performing the target behavior, which in this case is the distribution and explanation of PDIS. These domains were mapped into three COM-B components: nurses'capabilities (knowledge, skills, memory, and decision processes), motivations (role agreement, beliefs in PDIS consequences, beliefs in capabilities to implement, intentions, and goal setting), and opportunities (environmental context). The results have been published elsewhere and presented in detail in Additional file 2.

## Data analysis (stage 2 and 3): identify intervention options and content

An iterative process was undertaken by the research team with multiple meetings throughout the implementation intervention package development phase. Empirical data and relevant literature were continuously examined during these discussions. The primary focus of this stage was to determine which types of implementation interventions are most likely to address the determinants relating to PDIS implementation and what should be the specific implementation intervention content for the selected type of implementation interventions.

A paper-based exercise was conducted to map the COM-B components onto intervention functions, aligning with the BCW guideline [16]. The evaluation of each intervention function, utilizing the APEASE criteria [16] (Affordability, Practicability, Effectiveness and cost-effectiveness, Acceptability, Side effects and safety, and Equity), was independently carried out by the first author (DYXW). Potential alternative intervention functions were identified and presented during group meetings. Following this, iterative group discussions were initiated, involving two professionals with extensive clinical experience in nursing, medicine, and implementation science (ELYW and EKY) and two senior researchers engaged in the identification of the implementation determinants (AWLC and ZPYT). Each intervention function was subjected to thorough examination, and disagreements were resolved through discussions. The BCW framework encompasses seven comprehensive policy-level interventions, expected to be effective in supporting intervention functions. Similar to the assessment of intervention functions, the policy categories were assessed based on the APEASE criteria, and those perceived as most suitable to the local public inpatient care setting were carefully selected.

To specify the intervention content, first, the most frequently used behavior change techniques linked with our selected intervention functions were identified from the taxonomy of 93 behavior change techniques according to the BCW guidance [16]. Secondly, the appropriateness of each behavior change technique was evaluated based on the nurses’ comments obtained in stage 1, findings from literature reviews highlighting effective behavior change techniques in clinical behavior change implementation interventions in similar settings [10, 40], and considerations regarding the local context based on the APEASE criteria. The final selection of behavior change techniques was determined through group discussions. Subsequently, the intervention functions, along with the chosen behavior change techniques and broad-level policies, were transformed and operationalized into a comprehensive list of implementation intervention items.

## Data synthesis (stage 4): develop the logic model

The results were synthesized through the utilization of the IRLM framework [21]. In order to align with our findings, modifications were made to the original determinants category provided by IRLM from “intervention characteristics, inner setting, outer setting, characteristics of individuals, processes” to “capability, motivation, opportunity”. Subsequently, implementation interventions were filled in alongside the implementation determinants. To shed light on the underlying mechanisms of action through which these implementation interventions lead to the expected implementation outcomes, multiple classical theories and peer-reviewed literature were drawn upon [41–51]. The selection of implementation outcomes, service outcomes, and patient outcomes was guided by considerations of the local context, expert opinions (ELYW and EKY), and the existing research gap in implementation outcome studies [52].

## Results

### Identify intervention options and content

After conducting the mapping exercise, we identified five intervention functions that are most suitable for facilitating the implementation of PDIS: education, training, enablement, persuasion, and environmental restructuring, as summarized in Additional file 3. An intervention function would be excluded if three or more aspects of APEASE were uncertain or if any one aspect was not met. Specifically, education focuses on enhancing the nurses’ understanding of PDIS background, responsibilities, and positive outcomes. Training is to enhance operation skills in manipulating the PDIS system and communicating medication risks. Enablement is characterized by increasing means, such as involving pharmacists as aids to increase nurses’ capabilities to make PDIS explanations and establishing a bidirectional feedback platform to increase opportunities for information circulation. Persuasion through opinion leaders to foster positive feelings. Lastly, restructuring the physical environment by modifications to the PDIS design. Four intervention functions were considered unsuitable in the current context either due to potential unwanted side effects on nurses or because three or more aspects of APEASE were uncertain. Subsequently, four policy categories were identified: communication/marketing, service provision, environmental/social planning, and guidelines, as summarized in Additional file 4. Marketing through utilizing mass media to educate staff by delivering comprehensive background information related to PDIS. Additionally, communication was employed by involving opinion leaders in advocating the benefits of PDIS. The provision of diverse training services and the integration of referral services by pharmacists to assist in explaining medication side effects should be considered. Furthermore, it is crucial to plan the organizational environment to foster a culture of continuous learning and to refine the PDIS system accordingly. Lastly, the establishment of implementation guidelines is recommended to standardize processes and ensure consistency in PDIS implementation. Following this, eleven behavior change techniques were identified to enact each intervention function, which is summarized in Additional file 5. A multifaceted implementation intervention package was developed and shown in Tables 1 and 2. This implementation intervention package had four components: flexible and sustainable training, communication and marketing, environment redesign, and implementation guidelines. Table 3 presents the key features of each of the implementation intervention components with illustrative quotes from nurses.

**Table 1:** PDIS implementation strategy package to address barriers

No	Barriers	TDF domains	COM-B components	Selected intervention function	Selected policy categories	Selected BCTs	Translation of BCTs into PDIS implementation strategy items
1	1) PDIS drug database coverage is not enough; 2) Chinese version only is not enough;	Environmental context and resources	Physical opportunity	Environmental restructuring	Environmental/social planning	Restructuring the physical environment	Review the current discharge drug list in the medical department and renew the PDIS drug coverage; Create an English version of the PDIS form
2	Information flow about the PDIS project is not smooth between front-line users and program management level	Environmental context and resources	Physical opportunity	Enablement	Environmental/social planning	Restructuring the social environment	Establish a bidirectional platform to facilitate information exchange between nurses and the PDIS program committee
3	There is a time constraint when handling PDIS	Environmental context and resources	Physical opportunity	Enablement	Service provision	Social support (practical)	Establish a referral mechanism to direct inquiries to pharmacists at the dispensary to help explain medication side effects to patients or caregivers
4	PDIS is a not a priority when performing discharge education with multiple discharge materials on hand	Memory, attention, and decision process	Psychological capability	Education	Communication/marketing	Feedback on outcomes of behavior	Provide written positive outcomes related to both nurses and patients of the PDIS program to educate and persuade to create more positive beliefs about the PDIS program
				Enablement	Service provision	Social support (practical)	Establish a referral mechanism to direct inquiries to pharmacists at the dispensary to help explain medication side effects to patients or caregivers
					Environmental/social planning	Restructuring the physical environment	Incorporate the PDIS system into the Discharge Summary system
5	Training is not enough	Skills	Psychological capability	Training	Service provision	Demonstration of the behavior; Introduction on how to perform the behavior	Integrate PDIS training into the regular new staff orientation to demonstrate methods to execute PDIS on EHR and side effects explanations
						Restructuring the social environment	Set up a sharing group to encourage nurses to share their views and experience in terms of PDIS execution behaviors and techniques
			Psychological capability	Enablement	Environmental/social planning	Restructuring the physical environment	Establish a training system to continuously enhance PDIS-related medication knowledge and other necessary skills, such as interpersonal communication, to help nurses deliver the PDIS program

*Abbreviation*: *PDIS* Post-Discharge Information Summary; *TDF* Theoretical Domains Framework; *COM-B* Capability, Opportunity, Motivation, Behavior Model; *BCTs* Behavior Change Techniques

**Table 2:** PDIS implementation strategy package to enhance facilitators

No	Facilitators	TDF domains	COM-B components	Selected intervention function	Selected policy categories	BCTs	Translation of BCTs into PDIS implementation strategy items
1	Having knowledge regarding PDIS background, content, and role	Knowledge	Psychological capability	Education	Communication/marketing	Information about social and environmental consequences	Design brochures and make videos to introduce the PDIS program development background and objectives
					Guideline	Information about social and environmental consequences	Articulate job responsibilities and program content by formulating implementation guidelines
2	PDIS platform design is user-friendly	Environmental context and resources	Physical opportunity	Training	Service provision	Demonstration of the behavior; Introduction on how to perform the behavior	Integrate PDIS training into the regular new staff orientation to demonstrate methods to execute PDIS on EHR and side effects explanations
						Restructuring the social environment	Set up a sharing group to encourage nurses to share their views and experience in terms of PDIS execution behaviors and techniques
3	PDIS becomes routine	Memory, attention, and decision process	Psychological capability	Training	Service provision	Demonstration of the behavior; Introduction on how to perform the behavior	Integrate PDIS training into the regular new staff orientation to demonstrate methods to execute PDIS on EHR and side effects explanations
4	I think PDIS is useful for patients/caregivers and/or my work	Beliefs in consequences	Reflective motivation	Education; Persuasion	Communication/marketing	Feedback on outcomes of behavior	Provide written positive outcomes related to both nurses and patients of the PDIS program to educate and persuade to create more positive beliefs about the PDIS program
				Persuasion	Communication/marketing	Credible source	Invite leaders to convey the positive impact of the PDIS program and the importance of the role of nurses to induce positive emotions on role agreement Invite patients to give positive feedback to induce positive beliefs regarding PDIS values
5	I agree with my responsibility of PDIS	Social/professional role and identity	Reflective motivation	Education; Persuasion	Communication/marketing	Feedback on outcomes of behavior	Provide written positive outcome related to both nurses and patient of PDIS program to educate, persuade to create more positive beliefs about PDIS program
				Persuasion	Communication/marketing	Credible source	Invite leaders to convey the positive impact of the PDIS program and the importance of the role of nurses to induce positive emotions on role agreement
6	I am confident that I am able to implement the PDIS	Beliefs in capabilities	Reflective motivation	Education; Persuasion	Communication/marketing	Feedback on outcomes of behavior	Provide written positive outcome related to both nurses and patient of PDIS program to educate, persuade to create more positive beliefs about PDIS program
				Persuasion	Communication/marketing	Credible source	Invite peers to share positive experiences about PDIS program to induce positive beliefs about the ability to execute PDIS
7	Distributing PDIS to every discharged case is mandatory	Goals	Reflective motivation	Education; Persuasion	Communication/marketing	Feedback on outcomes of behavior	Provide written positive outcome related to both nurses and patient of PDIS program to educate, persuade to create more positive beliefs about PDIS program
				Persuasion	Communication/marketing	Credible source	Invite leaders to convey the positive impact of the PDIS program and the importance of the role of nurses to induce positive emotions on role agreement
8	I am willing to implement PDIS in the future	Intentions	Reflective motivation	Education; Persuasion	Communication/marketing	Feedback on outcomes of behavior	Provide written positive outcome related to both nurses and patient of PDIS program to educate, persuade to create more positive beliefs about PDIS program
				Persuasion	Communication/marketing	Credible source	Invite leaders to convey the positive impact of the PDIS program and the importance of the role of nurses to induce positive emotions on role agreement Invite patients to give positive feedback to induce positive beliefs regarding the consequences of PDIS program
9	I need constant practicing to implement PDIS	Skills	Psychological capability	Enablement	Environmental/social planning	Restructuring the physical environment	Incorporate the PDIS system into the Discharge Summary system
10	I can handle this task with my professional knowledge	Skills	Psychological capability	Enablement	Service provision	Demonstration of the behavior; Introduction on how to perform the behavior	Integrate PDIS training into the regular new staff orientation to demonstrate methods to execute PDIS on EHR and side effects explanations
						Social support (practical)	Establish a referral mechanism to direct inquiries to pharmacists at the dispensary to help explain medication side effects to patients or caregivers
					Guideline	Problem solving; Action planning	Establish a hospital-wide review panel to regularly assess PDIS implementation and formulate action plans to address potential execution challenges

*Abbreviation*: *PDIS* Post-Discharge Information Summary; *TDF* Theoretical Domains Framework; *COM-B* Capability, Opportunity, Motivation, Behavior Model; *BCTs* Behavior Change Techniques; *EHR* Electronic Health Record

**Table 3:** Key features of the strategy package

Components	Key features	Example quotations from nurses
Flexible and sustainable training services	Use hybrid formats	*“I think new staff will need training. Other staff doesn’t need unless there are program updates.”* *“I think internal communication is better than official training because the daily work won’t be interrupted.”*
Mass media and opinion leader campaigns	Make benefits visible	*“Maybe have to think about how to promote this program better. Because I rarely heard patients’ comments, I don't’ think this service is important or necessary. If we can see the benefits, we will have greater motivation.”*
	Frame the purpose	*“I think it’s important to explain clearly the reasons behind it, the concept, why there is this new project, the future development, *etc*.”*
Technology and workflow redesign	Upgrade to be compatible with current workflow	*“The drug database it out of date. Some medications were no longer prescribed and some new drugs are frequently prescribed now. About two-thirds of the medication are not covered. It’s important to regularly review and update the database”* *“From my perspective, I think the most important thing for discharge medication education is the what medication is changed, so it’s better for PDIS to include this part of information.”* *“As there are many ethnic minority patients coming in our hospital, currently there is only Chinese version. It’s hard for me to educate them without English version.”*
	Adopt a default design	*“Is it possible to modify the system to combine the PDIS with other discharge documents with one print button? If so, the work will be more efficient.”*
	Provide a digital option	*“Because of the Covid 19, we are comfortable with digital technologies. It would be more convenient if PDIS has a e-version, such as, incorporated into the patient portal.”*
	Multidisciplinary team approach	*“I think we can work with pharmacists. Some wards have resident pharmacists. I think we need pharmacists to help with explaining medication side effect.”* *“I think we can print the form and pharmacists can help with the detailed explanation when patients go to pharmacy to get their medication.”*
	Build up a shared-decision approach	*“I think there should be a mechanism involve front-line representatives from each cluster*^a^* participating in regular review of PDIS. Maybe set up a committee and send the feedback to the central government.”* *“I think it’s better to build up a platform to facilitate the collection of front-line users’ feedback in order to facilitate the refinement of the PDIS.”*
Corporate level audit & feedback	Concrete action plans	*“It would be better to clarify what parts I have to explain, and something I need to pay attention to.”*

*PDIS* Post Discharge Information Summary

^a^The public hospitals are divided into seven geographically diverse clusters in Hong Kong

## Develop the logic model

**Fig. 2:**
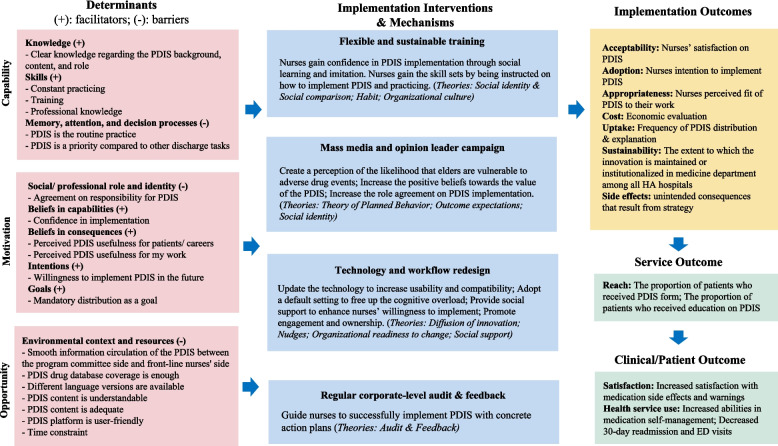
Theory of change of the PDIS implementation intervention package. Abbreviation: PDIS, Post-Discharge Information Summary; HA, Hospital Authority; ED, Emergency Department

The logic model was developed, illuminating the theory of change and theory of action underlying the implementation intervention package (Fig. 2). In the subsequent section, each component of the implementation intervention package will be explored, providing a detailed explanation of their potential to address implementation determinants and shed light on the underlying mechanisms leading to the expected implementation outcomes.

## Flexible and sustainable training

The behavior diagnosis highlighted the importance of nurses'professional knowledge in effectively explaining PDIS medication side effects. Additionally, the lack of integration of PDIS into routine discharge practice presents a challenge for implementation. To address this, the implementation intervention package includes the provision of training services to demonstrate the behaviors, aligning with commonly employed approaches for implementing evidence-based nursing [53–55]. Previous studies recommend that training should encompass diverse formats to accommodate different learning styles and staff adaptability to new technologies [56, 57]. Consequently, three levels of training were suggested. Firstly, new staff can receive PDIS training as part of their regular orientation, covering how to operate the PDIS system and explain medication side effects. Secondly, all nurses can engage in regular informal training through group sharing sessions, which have been shown to be highly effective for training purposes [58, 59]. These sessions facilitate reflection, debriefing, and sharing techniques to address challenges. Thirdly, establishing a training system allowing continuous staff training is proposed to ensure their PDIS skills remain up-to-date. Integrating training opportunities within a practical structure has been proven to increase the likelihood of behavior change [60]. Considering the time constraints frequently reported by nurses in clinical settings, a hybrid approach, which includes written materials, online learning, and face-to-face workshops, is suggested to provide a sustainable and convenient training method.

The first underlying mechanism can be elucidated through the concept of self-efficacy. When nurses possess the necessary skills, they tend to exhibit confidence and comfort in engaging with the PDIS process, thereby enhancing its acceptability and adoption. The second mechanism can be attributed to habit theory, wherein the successful integration of PDIS distribution and explanation into nurses’ routine practice becomes habitual. By automating this behavior through habit formation, cognitive capacity can be freed up amidst competing demands, potentially resulting in an increased uptake rate [41]. Thirdly, the establishment of sharing groups and a training system can cultivate an organizational culture that embraces change, which serves as a pivotal factor in modifying practitioner behavior [42, 43] and has the potential to contribute to PDIS sustainability.

## Mass media and opinion leader campaign

One of the implementation barriers is that nurses tend to prioritize alternative materials over PDIS when it comes to discharge education. However, several potential facilitators were identified, including positive beliefs about the benefits of PDIS for their work and patients, self-confidence in implementing PDIS, intention to implement, agreement on responsibilities, clear goals, and knowledge of the program background. To address these barriers and enhance facilitators, a range of communication and marketing strategies have been suggested. Notably, social norms interventions, which involve exposing healthcare workers to the values, beliefs, attitudes, or behaviors of reference individuals or groups, have shown effectiveness in multiple settings [61]. This can be achieved by engaging opinion leaders, including patients and peers, to advocate for PDIS and foster positive beliefs in PDIS values and implementation capabilities. Credible sources have been proven effective in changing health providers’ clinical behavior [61]. Combining reinforcement of new practice norms and peer group advocation is particularly effective as it establishes clear rules and encourages individuals to emulate their peers, according to a previous review [62]. Therefore, inviting nursing leaders to reinforce PDIS implementation and emphasize the importance of nurses as responsible individuals to induce positive emotions in role agreement is suggested. In order to make information visible, disseminating written reports to showcase the positive outcomes of PDIS for both nurses and patients can serve as educational and persuasive tools, promoting more positive beliefs. Lastly, the development of brochures and videos that effectively communicate the background and objectives of PDIS can enhance nurses'knowledge and understanding.

Social identity and social comparison serve as the mechanism of these implementation interventions [44, 45], wherein nurses evaluate their own abilities and compare themselves to their peers. This evaluation leads them to engage in behaviors that are congruent with the group norms and serve to maintain their social identity. Additionally, the theory of planned behavior [46] can explain prospective implementation outcomes as nurses are motivated to conform to the expectations of esteemed individuals, such as patients and leaders, in order to uphold their personal beliefs. The third mechanism is outcome expectations [10], whereby nurses perceive PDIS as beneficial for enhancing their workflow and improving patient outcomes to induce desired behaviors. These mechanisms have the potential to lead to a higher acceptability, adoption, appropriateness, and uptake rate.

## Technology and workflow redesign

The behavior diagnosis has revealed that the current design of the PDIS system hinders the implementation process for nurses. There is a lack of transparent information exchange between the program committee and frontline implementers. Time constraints also frequently arise as a barrier to implementation, particularly when there are multiple tasks to be completed. To address these issues, it is recommended, based on previous research, that technology-based interventions should complement the current workload of health providers and consider the diverse settings in which they operate [10]. Therefore, the PDIS system should be upgraded to better accommodate the needs of nurses, such as expanding the drug database and providing multiple language versions to facilitate their explanation. Furthermore, the use of default settings has been found to effectively change providers'behaviors in various contexts [63], particularly within electronic systems [64]. Therefore, incorporating PDIS information as a default setting in the discharge summary can alleviate cognitive overload for nurses who need to refer to multiple information sources for printing discharge forms. To facilitate smooth information flow, it is suggested that a bidirectional platform be established that facilitates communication and information exchange between nurses and the program committee. It’s emphasized that nurses should actively engage with information and communication technologies as managers and designers rather than having a passive relationship as mere users [65]. Additionally, a prior review highlighted the multidisciplinary team approach is crucial to integrating new interventions into routine practice, fostering collaborations, and leveraging specialized skills [66]. Therefore, establishing a referral mechanism to direct PDIS inquiries to the pharmacy, which can assist in explaining medication side effects to patients or caregivers, is recommended.

The first underlying mechanism aligns with the theory of diffusion of innovation [47], which suggests that improving the quality of PDIS design can positively influence perceived usefulness and subsequently impact the intention to use the technology. Secondly, drawing from the nudge theory in the field of behavioral economics [48], altering default choices to reduce the physical effort required to select among alternatives can facilitate uptake. Additionally, based on the theory of organizational readiness to change [49], end-user involvement is emphasized in effective implementation by increasing the change valence and realistically appraising the intervention fit. Lastly, social support serves as a mechanism characterized by creating a supportive environment to alleviate nurses'stress in explaining PDIS, thereby increasing the acceptability and uptake.

## Regular corporate-level audit and feedback

The behavior diagnosis has brought attention to the variation in nurses’ implementation due to inadequate knowledge regarding PDIS responsibility and its target population, resulting in implementation barriers. Furthermore, nurses with ample experience in discharge education and procedures are better equipped with clear action plans to effectively implement PDIS. Taking into account previous research that has highlighted the advantages of audit and feedback [51], as well as the effectiveness of action plans in enhancing the impact of such feedback [50], it is recommended to develop implementation guidelines that not only standardize the PDIS implementation process to educate nurses regarding their roles and PDIS content but also outline concrete contingency plans to address potential implementation challenges, accompanied by regular corporate-wide evaluations to ensure consistent implementation.

Initiating guidelines serves as the mechanism to regulate behaviors and promote adherence to desired practices. Guiding and directing individuals to solve implementation problems can enhance nurses’ self-efficacy and further increase possible adoption. An additional mechanism centers around applying audit and feedback techniques to impose external pressure, such as performance measures, to exert nurses’ motivation to perform desired behaviors.

## Discussion

Guided by BCW and IRLM, this study undertook a structured process of designing a theory-informed multifaceted implementation intervention package, aiming to enhance health providers’ implementation of a new information and communication technology for facilitating post-discharge self-management among hospitalized older adults. These components include the provision of flexible and sustainable training services to enhance nurses'skills, the utilization of opinion leaders and mass media for persuasive and educational purposes, the redesign of technology features and restructuring of the organizational environment, and the establishment of implementation guidelines that cover standardized procedures and concrete action plans. Each component was developed with explicit intervention functions, such as education, enablement, and persuasion. These functions were enacted through multiple behavior change techniques, including the use of credible sources, behavior demonstration, restructuring of the social environment, and problem-solving. Additionally, these components are supported by broader policies such as service provision, communication/marketing, and guidelines. The implementation intervention components are directly linked to desired implementation outcomes through the mechanisms of action underlying these components, which are drawn from various theories and facilitate future evaluation. By successfully achieving these implementation outcomes, it is anticipated that the implementation intervention will also lead to favorable service outcomes and clinical outcomes.

## Reflections on the BCW approach

It was confirmed by our study experience that the BCW framework proved to be valuable in formulating implementation interventions, particularly in addressing implementation determinants at the provider level. However, several pitfalls were identified that should be anticipated by practitioners and researchers engaged in implementation projects. Firstly, the mapping processes exhibited dynamic characteristics, with iterations occurring between the mapping of implementation determinants and intervention functions, as well as intervention functions and behavior change techniques during research team discussions to reach consensus on the appropriateness of the implementation intervention. Secondly, while BCW guidelines position policy category identification between intervention function identification and behavior change technique identification, it may be more logically placed after behavior change technique identification, as highlighted in previous studies that have applied the BCW [67, 68]. Thirdly, although the BCW allows for the specification of the smallest components of the implementation intervention, further attention should be given to operationalization details. For instance, the impact of dosage on implementation intervention effectiveness should be considered. Previous studies have indicated that delivering the implementation intervention once may be sufficient [61], particularly in the busy clinical environment, where allocation of time to support implementation competes with numerous demands [69]. The application of the Template for Intervention Description and Replication checklist can serve as a viable solution as it allows for a comprehensive consideration encompassing various aspects, including the underpinning theory, materials used, who provided, mode of delivery, location, timing, and dosage [70]. Finally, the TDF predominantly focus on individual and interpersonal factors, potentially overlooking broader organizational, systemic, and policy-level influences that can have an impact on behavior change. Organizational culture, socioeconomic situation, and policy directives are critical elements that may not be fully encapsulated within the framework’s domains. This underscores the need for an approach that incorporates additional models or frameworks to provide a more comprehensive understanding of the influential factors. For example, several studies combined the TDF with the Consolidated Framework for Implementation Research [71] to explore the multi-level nature of the implementation processes [72].

In addition, as the BCW was developed within a Western context, it is important to apply it with caution when applying it to the Eastern Asia area such as Hong Kong with a different cultural context and healthcare system. The APEASE criteria is especially useful when selecting the potential intervention functions and policy categories as we can take our cultural context into consideration to see the feasibility and acceptability and make cultural adapted and fit decision towards the final list of implementation interventions.

As we proceed to test the implementation interventions developed in our study, we recognize that while the BCW offers a detailed understanding of behavioral determinants and aids in implementation intervention development, it does not provide guidance on formulating hypotheses to test the effectiveness of these implementation interventions. The IRLM complements this by enhancing planning and evaluation, allowing for a systematic assessment of implementation interventions. It helps to identify implementation outcomes and the mechanisms through which implementation interventions may lead to these outcomes [73]. Integrating the IRLM with the BCW allows us to clearly articulate the program theory, facilitating communication among stakeholders through a standardized structure. This combined approach can enhance the overall efficiency of our research efforts.

## Strengths and limitations

During the process of developing the implementation intervention package, several strengths were identified. Firstly, the detailed recording of multiple available options and the application of APEASE criteria enhanced the transparency and generalizability of the methods by illustrating the reasoning behind the selection or exclusion of options. Secondly, the mapping process facilitated the evaluation of each implementation intervention component's effectiveness by referring to the logic model. Lastly, considering the scarce evidence on the effectiveness of implementation theory-informed implementation intervention design [53], our study facilitates the comparison of the benefits between implementation interventions with and without implementation theory. However, limitations exist when employing this approach. Firstly, the application of the BCW approach has been recognized to have a specific weakness wherein the absence of designated chances for stakeholder involvement, which is considered a fundamental principle in modern healthcare, is acknowledged [74]. To address this, a Delphi expert discussion will be conducted in the next step to formulate contextually adapted implementation interventions, ensuring expert consensus on their relevancy, acceptability, and feasibility within the Hong Kong public healthcare setting. Secondly, adhering to all steps listed in the BCW guideline consumed a substantial amount of time, especially during the behavior diagnosis phase. However, this meticulous approach ensured the consideration of all potential implementation determinants and implementation intervention components for effective behavior change. Future research could explore the use of rapid assessment procedures [75], which employ a team approach and iterative data collection and analysis to produce contextually rich evaluative information within shorter timelines, typically spanning weeks to months from start to finish.

## Implications for future research and implementation practice

Given the complexity of our implementation intervention package, delivering all components simultaneously is not feasible. To optimize the use of resources, the Multiphase Optimization Strategy framework offers a valuable approach for determining the most effective combination and sequence of components or items to achieve optimal implementation outcomes [76]. This three-step framework involves screening the isolated effect of each component, refining effective components through experimentation to identify the optimal level of exposure, and validating the final version through a standard randomized control trial. Additionally, Adaptive Interventions and the Sequential Multiple Assignment Randomized Trial can be applied [76] in conjunction with a pre-implementation evaluation at the hospital level, allowing for the customization of components based on individual and organizational characteristics. This iterative process involves step-wise adaptation of the implementation intervention and continuous assessment of the variables. The IRLM can be employed to streamline this specific design by categorizing the status of each implementation intervention component into four types [21]: (i) already in place prior to the study, (ii) initiated prospectively for the study, (iii) removed due to ineffective or onerous, and (iv) introduced during the study to address emerging barriers or complement other implementation interventions.

The comprehensive documentation of our implementation intervention development process serves as a valuable resource for researchers seeking to develop their own information and communication technology implementation interventions. It highlights critical considerations to which they should pay close attention. Given the rapid evolution inherent to technology, it is essential to adopt a dynamic and iterative approach when implementing information and communication technologies in clinical setting. Our study rigorously adhered to all steps of the BCW approach. However, the COVID- 19 pandemic imposed significant challenges, particularly in conducting individual interviews, resulting in an around three-year timeline to design the implementation intervention. This duration may lead to discrepancies with the most current clinical needs of nurses. Therefore, we recommend that researchers and implementation practitioners prospectively plan for successful information and communication technology implementation by conducting pre-implementation and ongoing assessments of contextual factors, and allowing flexibility in implementation intervention design by identifying core components and permitting adaptability in others. Additionally, we advocate for building implementation capacity among clinical staff to empower them to proactively engage in planning and evaluation during implementation, given their firsthand experience and ability to respond swiftly to issues.

In addition, our implementation intervention development process embraced the concept of co-creation, incorporating nurses'suggestions for enhancing the implementation process into the selection and operationalization phase of behavior change techniques. Engaging stakeholders in healthcare initiatives is strongly recommended [77]. This inclusion is vital to enhance the chances of successful implementation and ensure the feasibility of the developed implementation interventions [78]. While some implementation intervention developers engage stakeholders throughout the development process (e.g., selecting intervention functions, policy categories, and behavior change techniques) [79, 80], our approach offers efficiency gains in research efforts. Recognizing the limited familiarity with implementation science concepts among clinical staff, we simplified our inquiries to focus on their perceptions of barriers and facilitators and ways for process enhancement. This approach not only reduces cognitive load but also logically elicits suggestions based on their reflections on implementation issues. To further ensure that the designed implementation interventions align with their preferences, we plan to conduct a Best Worst Scaling survey to capture their priorities for implementation interventions [81].

## Conclusions

The utilization of technology by health providers in their clinical practice is increasingly recognized, yet the current evidence base for implementation interventions supporting the clinical implementation of information and communication technologies remains incomplete. In order to address this gap, this study employed a systematic approach to develop a theory-informed multifaceted implementation intervention package aiming to improve health providers'implementation of a new information and communication technology designed to facilitate post-discharge self-management among hospitalized older adults. By offering a worked example of the application of the BCW approach in conjunction with the logic model, this study contributes to the existing knowledge base on the design of implementation interventions. Furthermore, the findings of this study can serve as a valuable reference for organizations seeking to improve health providers'behavior change and enhance the overall quality of post-discharge care management, particularly through the utilization of technology-based interventions.

## Supplementary Information

Additional file 1.

Additional file 2.

Additional file 3.

Additional file 4.

Additional file 5.

## Data Availability

Data generated or analyzed during this study are included in this published article and its supplementary information files. Additional datasets are available from the corresponding author on reasonable request.

## Abbreviations

PDIS: Post Discharge Information Summary
BCW: Behavior Change Wheel
TDF: Theoretical Domains Framework
COM-B model: Capability, Opportunity, Motivation, Behavior model
IRLM: Implementation Research Logic Model
