# Supplementary material for: Enhancing implementation of information and communication technologies for post-discharge care among hospitalized older adult patients: development of a multifaceted implementation intervention package using the behavior change wheel and implementation research logic model
Source: Implement Sci Commun. 2025 May 1;6:52. doi: 10.1186/s43058-025-00739-4 (PMC12046763; doi:10.1186/s43058-025-00739-4)
Supplement: Supplementary file 2 — Additional file 2. [file 43058_2025_739_MOESM2_ESM.docx]

**Additional file 2. PDIS distribution & explanation determinants**

| **COM-B Component** | **TDF Domains** | **Target behavior** | |
| --- | --- | --- | --- |
|  |  | **Distribution** | **Explanation** |
| Physical capability | Skills | - I need constant practicing to print PDIS | - Training helps me with explaining PDIS content - I can handle this task with my professional knowledge |
| Psychological capability | Knowledge | - I have knowledge regarding the PDIS objective (development path, program purpose, and goal) - I have knowledge regarding the PDIS target population | - I have knowledge regarding the PDIS objective (development path, program purpose, and goal) - I clearly know explaining PDIS is part of my responsibility |
|  | Memory, attention, and decision processes | - Distributing PDIS is my routine practice - PDIS is a priority when performing discharge education with multiple discharge materials on hand | - Explaining PDIS is my routine practice - PDIS is a priority when performing discharge education with multiple discharge materials on hand |
| Reflective motivation | Social/ professional role and identity | - I agree with my responsibility of printing and distributing PDIS - PDIS representative is a key person in program implementation | - I agree with my responsibility of explaining PDIS content - PDIS representative is a key person in program implementation |
|  | Beliefs about capability | - I am confident that I am able to print the PDIS | - I am confident that I am able to explain the PDIS content |
|  | Beliefs about consequences | - I think PDIS is useful for patients/ careers - I think PDIS is useful for my work | - I think PDIS is useful for patients/ careers - I think PDIS is useful for my work |
|  | Intentions | - I am willing to use PDIS to perform discharge education in the future | - I am willing to instruct medication side effects in the future |
|  | Goals | - Distributing PDIS to every discharged case is mandatory | N/A |
| Physical opportunity | Environmental context and resources | - Language versions are not enough for current patient population - PDIS platform design is user-friendly - There is time constraint when printing PDIS | - Information sharing of PDIS is not quick and enough between program committee and front-line staff - Information on PDIS is clear to patients/ caregivers - Drug database is out of date - Information coverage of PDIS is right enough for me to educate patients/ caregivers - There is time constraint when explaining PDIS |

TDF: Theoretical Domains Framework; PDIS: Post Discharge Information Summary
