# Supplementary material for: Enhancing implementation of information and communication technologies for post-discharge care among hospitalized older adult patients: development of a multifaceted implementation intervention package using the behavior change wheel and implementation research logic model
Source: Implement Sci Commun. 2025 May 1;6:52. doi: 10.1186/s43058-025-00739-4 (PMC12046763; doi:10.1186/s43058-025-00739-4)
Supplement: Supplementary file 3 — Additional file 3. [file 43058_2025_739_MOESM3_ESM.docx]

**Additional file 3 Linking gaps in empiric data for behavior diagnosis to intervention function and appraisal with APEASE criteria**

| **Barrier/ Facilitator** | **TDF Domains** | **COM-B Component** | **BCW Intervention Functions** | **Does it meet APEASE criteria** | **Decision***  **Yes/No** |
| --- | --- | --- | --- | --- | --- |
| -Knowledge regarding PDIS background  -Knowledge regarding PDIS content  -Knowledge regarding PDIS role | Knowledge | Psychological capability | Education | Yes | Y |
|  |  |  | Training | Unrelated | N |
|  |  |  | Enablement | Unrelated | N |
| -Information circulation of the PDIS program is enough and smooth between the program committee/ working group side and front-line staff side  -Information on PDIS is clear to patients/ caregivers  -PDIS drug database coverage is not enough  -The Chinese version is good enough for the current patient population  -Information coverage of PDIS is right enough for me to educate patients/ caregivers  -PDIS platform design is user-friendly  -There is a time constraint when handling PDIS  -Covid 19 impacts the implementation of PDIS | Environmental context and resources | Physical opportunity | Environmental restructuring | Practicability: not sure whether it would be practical to renew the drug entities and amend the PDIS form. Effectiveness: this is uncertain but judged to be worth evaluating | Y |
|  |  |  | Enablement | Practicability: not sure whether it would be practical to involve pharmacists and set up a two-way feedback system to enhance information dissemination due to manpower constraints.  Effectiveness: this is uncertain but judged to be worth evaluating | Y |
|  |  |  | Restriction | Practicability and acceptability: not sure whether setting rules to spend a certain time to conduct PDIS is practical and acceptable to nurses and hospitals;  Side effects: if strict rules are set, nurses will have negative feelings. Effectiveness: this is uncertain but judged to be worth evaluating | N |
|  |  |  | Training | Practicability: not sure whether all nurses could attend due to busy clinical environment. | Y |
| -PDIS is my routine practice  -PDIS is a priority when performing discharge education with multiple discharge materials on hand | Memory, attention, and decision processes | Psychological capability | Education | Yes | Y |
|  |  |  | Training | Practicability: not sure whether all nurses could attend due to busy clinical environment. | Y |
|  |  |  | Enablement | Practicability: not sure whether it would be practical to involve pharmacists due to manpower constraints.  Effectiveness: this is uncertain but judged to be worth evaluating | Y |
| -I think PDIS is useful for patients/ careers  -I think PDIS is useful for my work | Beliefs in consequences | Reflective motivation | Education | Yes | Y |
|  |  |  | Persuasion | Opinion leaders campaign for the positive impact of PDIS to induce positive emotions.  Effectiveness: this is uncertain but judged to be worth evaluating | Y |
|  |  |  | Incentivization | Affordability: not sure whether the cost could be covered.  Practicability and acceptability: not sure whether it would be practical and acceptable to the hospital management level. | N |
|  |  |  | Coercion | Acceptability: not sure whether it would be acceptable to nurses  Side effects: it is easy to cause unwanted side effects with punishment | N |
| -I agree with my responsibility of PDIS | Social/ professional role and identity | Reflective motivation | Education | Please refer to “Beliefs in consequences” domain | Y |
|  |  |  | Persuasion |  | Y |
|  |  |  | Incentivization |  | N |
|  |  |  | Coercion |  | N |
| -I am confident that I am able to implement the PDIS | Beliefs in capability | Reflective motivation | Education | Please refer to “Beliefs in consequences” domain | Y |
|  |  |  | Persuasion |  | Y |
|  |  |  | Incentivization |  | N |
|  |  |  | Coercion |  | N |
| -Distributing PDIS to every discharged case is mandatory | Goals | Reflective motivation | Education | Please refer to “Beliefs in consequences” domain | Y |
|  |  |  | Persuasion |  | Y |
|  |  |  | Incentivization |  | N |
|  |  |  | Coercion |  | N |
| -I am willing to implement PDIS in the future | Intentions | Reflective motivation | Education | Please refer to “Beliefs in consequences” domain | Y |
|  |  |  | Persuasion |  | Y |
|  |  |  | Incentivization |  | N |
|  |  |  | Coercion |  | N |
| - I need constant practicing to implement PDIS  -Training helps with the implementation of PDIS  -I can handle this task with my professional knowledge | Skills | Psychological capability | Education | Not related to skills domain | N |
|  |  |  | Training | Practicability: not sure whether all nurses could attend due to busy clinical environment. | Y |
|  |  |  | Enablement | Practicability: not sure whether it would be practical to involve pharmacists due to manpower constraints.  Effectiveness: this is uncertain but judged to be worth evaluating | Y |

*Decision rules: intervention functions will not be included if >=3 question mark on any of the APEASE appraisal criteria, or >=1 “N” mark on any of the APEASE appraisal criteria. “Y” denotes “meet the criterion, “N” denotes “do not meet the criterion”, “?” denotes “not sure”, “N/A” denotes “Not Applicable
