# Supplementary material for: Enhancing implementation of information and communication technologies for post-discharge care among hospitalized older adult patients: development of a multifaceted implementation intervention package using the behavior change wheel and implementation research logic model
Source: Implement Sci Commun. 2025 May 1;6:52. doi: 10.1186/s43058-025-00739-4 (PMC12046763; doi:10.1186/s43058-025-00739-4)
Supplement: Supplementary file 4 — Additional file 4. [file 43058_2025_739_MOESM4_ESM.docx]

**Additional file 4** **Linking intervention functions to policy categories and appraisal with APEASE criteria**

| **Intervention Functions** | **Policy category** | **Does it meet APEASE criteria** | **Decision**  **Yes/ No*** |
| --- | --- | --- | --- |
| Education | Communication/ marketing | Effectiveness is uncertain but judged to be worth evaluating | Y |
|  | Guideline | Effectiveness is uncertain but judged to be worth evaluating | Y |
|  | Regulation | Acceptability: seems not acceptable for nurses. | N |
|  | Legislation | Not applicable | N |
|  | Service provision | Not related | N |
| Environmental restructuring | Guideline | Not related | N |
|  | Fiscal measures | Not applicable | N |
|  | Regulation | Not related | N |
|  | Legislation | Not applicable | N |
|  | Environmental/ social planning | PDIS form refinement.  Practicability: not sure whether it is practical in terms of technology issues.  Effectiveness is uncertain but judged to be worth evaluating | Y |
| Enablement | Guideline | Effectiveness is yet to test. | Y |
|  | Fiscal measures | Not applicable | N |
|  | Regulation | Acceptability: setting rules seem not acceptable for nurses | N |
|  | Legislation | Not applicable | N |
|  | Environmental/ social planning | Practicability: not sure whether it is practical in terms of resource constraints and technology issues.  Effectiveness: this is uncertain but judged to be worth evaluating. | Y |
|  | Service provision | Practicability: not sure whether it is practical in terms of manpower constraints.  Effectiveness: this is uncertain but judged to be worth evaluating. | Y |
| Training | Guideline | Not related | N |
|  | Fiscal measures | Not applicable | N |
|  | Regulation | Not related | N |
|  | Legislation | Not applicable | N |
|  | Service provision | Practicability: not sure whether all nurses could have time to attend. | Y |
| Persuasion | Communication/ marketing | Effectiveness: this is uncertain but judged to be worth evaluating. | Y |
|  | Guideline | Not related | N |
|  | Regulation | Not related | N |
|  | Legislation | Not applicable | N |
|  | Service provision | Not related | N |

*Decision rules: policy categories will not be included if >=3 question mark on any of the APEASE appraisal criteria, or >=1 “N” mark on any of the APEASE appraisal criteria. “Y” denotes “meet the criterion, “N” denotes “do not meet the criterion”, “?” denotes “not sure”, “N/A” denotes “Not Applicable”
