# Supplementary material for: Enhancing implementation of information and communication technologies for post-discharge care among hospitalized older adult patients: development of a multifaceted implementation intervention package using the behavior change wheel and implementation research logic model
Source: Implement Sci Commun. 2025 May 1;6:52. doi: 10.1186/s43058-025-00739-4 (PMC12046763; doi:10.1186/s43058-025-00739-4)
Supplement: Supplementary file 5 — Additional file 5. [file 43058_2025_739_MOESM5_ESM.docx]

**Additional file 5 Link intervention functions to behavior change techniques and appraisal APEASE to identify potentially relevant behavior change techniques**

| **Intervention Functions** | **Most frequently used behavior change techniques** | **Does the behavior change technique meet APEASE criteria** | **Decision (Yes/ No)*** |
| --- | --- | --- | --- |
| Education | 5.3 Information about social and environmental consequences; | Yes | Y |
|  | 5.1 Information about health consequences | Yes | Y |
|  | 2.2 Feedback on behavior | Not related | N |
|  | 2.7 Feedback on outcomes of behavior | Yes | Y |
|  | 7.1 Prompts/ cues | Unlikely to be effective | N |
|  | 2.3 Self- monitoring of behavior | Unlikely to be acceptable | N |
| Environmental restructuring | 12.1 Restructuring the physical environment | Not sure whether it is practical in terms of technology issues  Effectiveness is uncertain but judged to be worth evaluating | Y |
|  | 12.5 Adding objects to the environment | Not related | N |
|  | 7.1 Prompts/ cues | Not related | N |
| Enablement | 6.1 Demonstration of the behavior | Not related | N |
|  | 3.2 Social support (practical) | Not sure whether it is practical in terms of technology issues  Effectiveness is uncertain but judged to be worth evaluating | Y |
|  | 3.1 Social support (unspecified) | Not related | N |
|  | 1.2 Problem solving | Yes | Y |
|  | 1.4 Action planning | Yes | Y |
|  | 1.1 Goal setting (behavior) | Unlikely to be acceptable for nurses | N |
|  | 1.3 Goal setting (outcome) | Not related | N |
|  | 12.5 Adding objects to the environment | Not related | N |
|  | 2.3 Self- monitoring of behavior | Not related | N |
|  | 12.2 Restructuring the physical environment | Not sure whether it is practical regarding technology issues. | Y |
|  | 12.2 Restructuring the social environment | Not sure whether it is practical in terms of resource constraints.  Effectiveness is uncertain but judged to be worth evaluating. | Y |
|  | 1.5 Review behavior goal | Not related | N |
|  | 1.7 Review outcome goal | Not related | N |
| Training | 6.1 Demonstration of the behavior | Practicability: not sure whether all nurses could have time to attend. | Y |
|  | 4.1 Introduction on how to perform the behavior | Practicability: not sure whether all nurses could have time to attend. | Y |
|  | 2.2 Feedback on behavior | Not related | N |
|  | 2.7 Feedback on outcomes of behavior | Not related | N |
|  | 2.1 Monitoring of behavior by others without feedback | Unlikely to be acceptable | N |
|  | 2.5 Monitoring of outcome by others without feedback | Not related | N |
|  | 2.3 Self- monitoring of behavior | Not related | N |
| Persuasion | 9.1 Credible source | Yes | Y |
|  | 5.3 Information about social and environmental consequences; | Not related | N |
|  | 5.1 Information about health consequences | Not related | N |
|  | 2.2 Feedback on behavior | Not related | N |
|  | 2.7 Feedback on outcomes of behavior | Yes | Y |

*Decision rules: Behavior change techniques will not be included if >=3 question mark on any of the APEASE appraisal criteria, or >=1 “N” mark on any of the APEASE appraisal criteria. “✓” denotes “meet the criterion, “N” denotes “do not meet the criterion”, “?” denotes “not sure”, “NA” denotes “Not Applicable”
